# Thermal illumination limits in 3D Raman microscopy: A comparison of different sample illumination strategies to obtain maximum imaging speed

**DOI:** 10.1371/journal.pone.0220824

**Published:** 2019-08-13

**Authors:** Walter Hauswald, Ronny Förster, Jürgen Popp, Rainer Heintzmann

**Affiliations:** 1 Institute of Physical Chemistry and Abbe Center of Photonics, Friedrich Schiller University Jena, Jena, Germany; 2 Leibniz Institute of Photonic Technology, Jena, Germany; Nicolaus Copernicus University, POLAND

## Abstract

Confocal Raman microscopy is a powerful tool for material science and biomedical research. However, the low Raman scattering cross-section limits the working speed, which reduces the applicability for large and sensitive samples. Here, we discuss the fundamental physical limits of Raman spectroscopy with respect to signal-to-noise, sample load and how to achieve maximal imaging speed. For this, we develop a simple model to describe arbitrary far field light microscopes and their thermal influence on the sample. This model is used to compare the practical applicability of point- and line-confocal microscopes as well as wide-field-, light sheet- and light line illumination, for the measurement of 3D biological samples. The parallelization degree of the illumination can positively affect the imaging speed as long as it is not limited by thermal sample heating. In case of heat build-up inside the sample, the advantages of parallelization can be lost due to the required attenuation of excitation and the working speed can drop below that of a sequential method. We show that for point like illumination, the exposure time is thermally not as critical for the sample as the irradiance, while for volume like illumination, the exposure time and irradiance result in the same thermal effect. The results of our theoretical study are experimentally confirmed and suggest new concepts of Raman microscopy, thus extending its applicability. The developed model can be applied to Raman imaging as well as to other modes (e.g. two- or three- photon imaging, STED, PALM/STORM, MINFLUX) where thermal effects impose a practical limit due to the high irradiance required.

## Introduction

Confocal Raman microscopy [1] reveals a multidimensional chemical image contrast of samples without the need of prior labelling or staining. Spontaneous Raman scattering is, thus, a powerful analytical tool for all kind of natural research like e.g. material science, biomedical research or medical diagnostics [2,3]. However the low Raman scattering cross-section [4] and the sensitivity of especially biological samples to the excitation light limits the speed, which makes live-cell imaging challenging. Since fluorescence- or elastic scattering-based imaging yields a signal that is orders of magnitude stronger, these methods are much faster and therefore often the method of choice, even though their contrast is much less revealing. Here, we performed a detailed theoretical study to understand the fundamental physical speed limitations of various imaging schemes especially in the context of Raman microscopy. It results in a new perspective on Raman microscopy serving as a guide to achieve maximal image speed.

To collect useful spatial information about a 2-dimensional (2D) or 3-dimensional (3D) sample, it is required to achieve a suitable contrast with sufficient image quality in an acceptable acquisition time while not influencing or damaging the sample. Sufficient image quality is associated with an appropriate image resolution and an adequate signal-to-noise ratio (*SNR*). All methods of light microscopy suffer from the mutual trade-off between image quality, acquisition time and effect on the sample.

The maximal speed in light microscopy is, apart from possible technical limitations, often given by a maximal acceptable local irradiance. In linear fluorescence imaging, a sensible limit to the irradiance is ultimately set by the saturation-limit (e.g. 4·10^−4^ mW·μm^-2^ for Venus at 532 nm [5]). Irradiance beyond this value does not yield linearly more emission signal due to the finite fluorescence-lifetime in the nanosecond range. For nonlinear fluorescence, as it is utilized in super-resolution microscopy, the irradiance limit depends additionally on wavelength, exposure time, labelling and sample. It becomes even more critical for live cell investigations [6]. The Raman scattering process features a much shorter life-time than fluorescence, directly allowing for a significantly increased local irradiance, which is in practice needed to compensate for the low scattering cross section (e.g. 140 mW·μm^-2^ at 532 nm [7]). However, the physical speed-limit to Raman microscopy is given by the sample being excessively heated, which has to be avoided. The temperature rise is determined by local linear absorption of the sample and the embedding medium in combination with their ability to dissipate thermal energy. With this, the maximal speed in Raman microscopy is not only limited by local conditions but by the heat build-up in the whole sample. This leads to the presumption that a careful design of illumination beyond a single-beam confocal scheme may optimise and accelerate the acquisition process.

There are many approaches of parallelization to accelerate Raman microscopy [8–13], some of them being implemented in commercial systems. Unfortunately, the maximal achievable acquisition speed does not simply scale linearly with the degree of parallelization. This is due to thermal interaction. The heat from a single spot can easily dissipate to the surrounding, whereas the heat of a large volume cannot. In contrast, in linear fluorescence imaging, parallelization can be assumed to lead to a proportional increase in speed since the singlet state saturation usually limits the maximal exposure prior to excessive heating.

In some Raman microscopes the irradiance is constrained for technical reasons or intentionally [10] to a low, fixed value. In such systems excessive sample heating caused by simultaneous illumination can be neglected and a large speedup due to parallelization can thus be obtained. However, if one attempts to image at maximal speed without reaching a temperature threshold where the sample would be modified [14] or even destroyed, it is hard to predict which scheme performs best, since the heat transfer inside the sample plays a major role.

**Fig 1:**
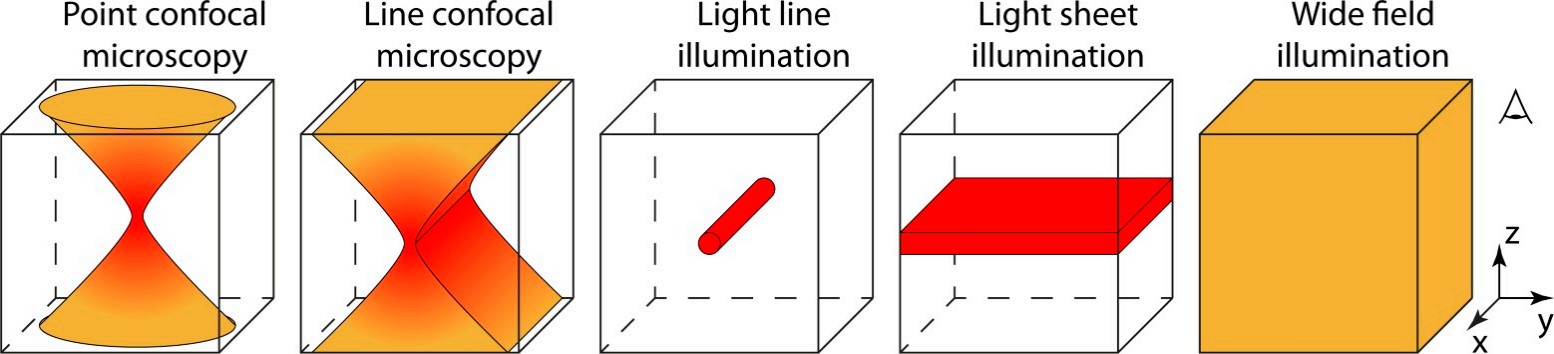
Five different illumination geometries common for light microscopy.

Since the accumulation of illumination-induced heat in the sample defines the main practical speed limit to Raman microscopy, we set out to study its dependence on various factors. For this, we develop a simple model describing the temperature rise and image *SNR* achieved by far field light microscopes based on various illumination geometries and data acquisition strategies. This model is used to compare the achievable *SNR* for a given acquisition time and for a given permissible temperature rise. The investigated acquisition schemes varying in illumination geometry are: point- [1,15] and line-confocal [8–10] microscopes as well as light line-, light sheet- [16–21] and wide-field illumination (**Fig 1**). We have chosen these five illumination types as possible geometrical extreme cases. The scanning strategy can be neglected in terms of imaging speed under conditions which are discussed together with the heat diffusion. The possible need for hyperspectral data acquisition is first neglected but its influence on the image *SNR* is considered in the results section. Our model can be applied to Raman imaging as well as to other modes (e.g. two- or three- photon imaging [22], STED [23], PALM [24]/STORM [25], MINFLUX [26]) where thermal effects cause a practical limit due to the high irradiance required.

The theory-heavy “materials and methods” section is followed by application-oriented results and a discussion, which might be interesting for some readers to consider before reading the part: “Signal yield of the generalised microscope”.

## Materials and methods

### A generalised optical microscope

**Fig 2:**
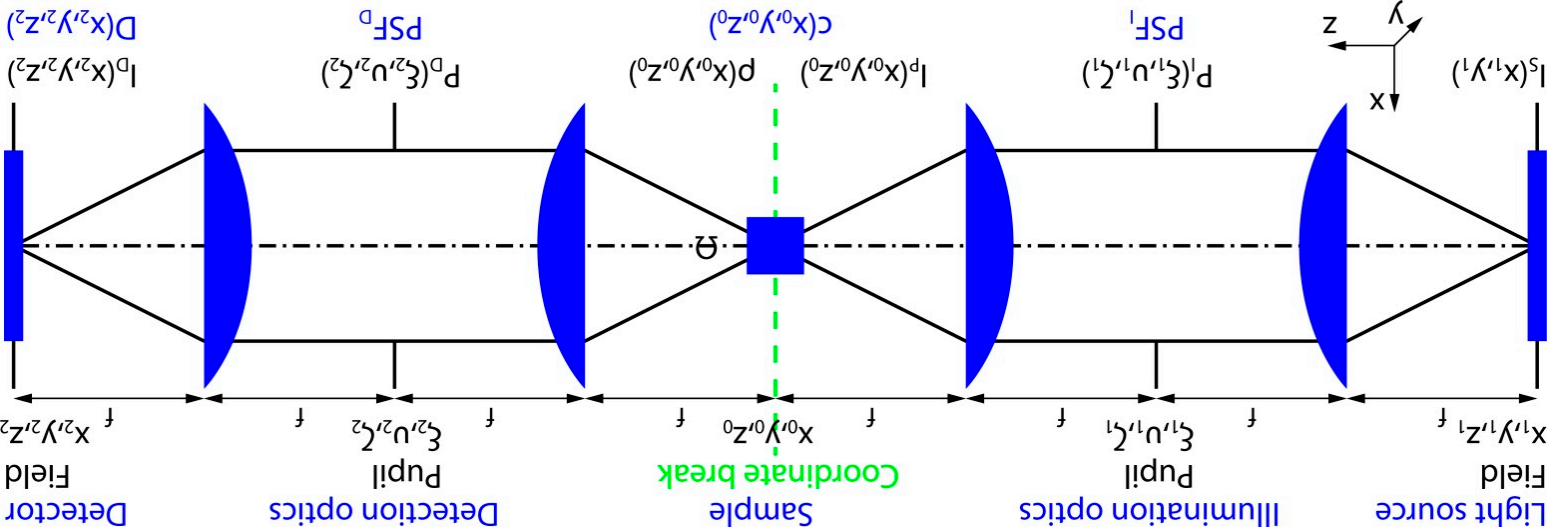
Generalised optical microscope. Illumination and detection optics can be at a relative angle. Without loss of generality, the magnification is assumed to be equal to one.

A generalised optical microscope [27] consists of a light source, illumination optics, a sample, detection optics and a detector (**Fig 2**). More general than shown in **Fig 1**, illumination and detection optics do not necessarily share a common optical axis but can be at a relative angle (e.g. in light-sheet geometry or in epi-illumination geometry as in most fluorescent and Raman setups).

Crucial for a mathematical description of an imaging system are the properties of the contrast-generating mechanism. Because spontaneous Raman scattering yields (like fluorescence emission) incoherent light, the imaging system can be described in terms of an incoherent point spread functions (PSF) [1,28]. Crucial for a mathematical description of the temperature distribution inside the sample is the heat dissipating mechanism. Since water is rather opaque (*μ*_*abs*_ ≈ 0.1 μm^-1^) [29] for thermal radiation (> 13.6 μm) at typical temperatures (< 373 K) and flow in the absence of vessels is small, we consider heat diffusion only.

Using this scheme, we calculate first the signal yield of the microscope and second the heat distribution in the sample. The signal yield on the one hand will enable us to calculate the achievable *SNR* as a function of irradiance. The heat distribution in the sample on the other hand will result in an illumination geometry-dependent temperature factor linking the exposure time and the irradiance in a way that a fixed temperature threshold is never exceeded. By combining both, the irradiance-dependent *SNR* with the temperature-dependent maximal irradiance, we obtain a maximal achievable *SNR* only depending on the permitted exposure time for each system.

The absolute *SNR* will, of course, depend on many factors like: Raman scattering cross-section of the sample, absorption coefficient of the sample, concentration of Raman scatterers, apparent pixel size, and the maximal permissible temperature rise, but these are independent of the instrument and therefore do not affect our relative comparison of illumination geometries. Therefore, we normalize the *SNR* by all of these factors and introduce the relative signal-to-noise ratio: *SNR*_*N*_.

### Signal yield of the generalised microscope

In Raman imaging the irradiance *I*_*D*_ at the detector associated with the scan- or pixel position *r*_*s*_ is approximated by:

$$I_{D}\left({\vec{r}}_{s}\right)=\left[\left(I_{P}\cdot \eta \cdot PSF_{D}\right){⊗}_{3}\rho \right]\;\left({\vec{r}}_{s}\right)$$ (1)

Here *I*_*P*_(*r*_*0*_) is the illumination distribution inside the sample, *PSF*_*D*_ is the detection point spread function and the constant *η* ≤ 1 accounts for detection losses (**Fig 2**). The operator ⊗_3_ is referring to the 3-dimensional convolution. The local ability of the sample to emit under illumination *ρ*(*r*_*0*_) is given by the product of the Raman scattering cross-section *σ*_*R*_ for a single molecule (e.g. 7.5 · 10^-30^ cm^2^ for water at 500 nm [4]), the concentration distribution of Raman scatterer *c*(*r*_*0*_) and the Avogadro constant *N*_*A*_:

$$\rho \left({\vec{r}}_{0}\right)=\sigma_{R}\cdot c\left({\vec{r}}_{0}\right)\cdot N_{A}$$ (2)

To compare the signal yield of different microscopes, we now set ρ to a spatially uniform distribution, being only for line-confocal and wide-field microscopy confined in depth (along z see **Fig 1**). Thus, the convolution in Eq 1 becomes independent of *r*_*s*_:

$$I_{D}=\eta \cdot \sigma_{R}\cdot N_{A}\cdot c\int \int \int I_{P}\left({\vec{r}}_{0}\right)\cdot PSF_{D}\left({\vec{r}}_{0}\right)d{\vec{r}}_{0}.$$ (3)

In confocal microscopy, the size of the detection pinhole affects the image quality, the detection efficiency and, with the latter, also the working speed. Thus, a comparison of different microscope geometries requires the consideration of this parameter, too. Since the relative detection efficiency of confocal microscopes is already discussed in detail elsewhere [1,30,31], we consider here the idealized point detector *D*(*r*_*2*_) with an integral efficiency of one.

To proceed with an analytical description of the optical system it is convenient to consider Gaussian pupils *P*_*I*_ and *P*_*D*_ (**Fig 2**) and to utilize (paraxial) Gaussian optics. Although this assumption works best with low numerical aperture (NA) optics, it yields sufficient results for our comparison. The irradiance distribution of an elliptic Gaussian beam [32] with propagation in z-direction is given by:

$$I_{P}\left(x,y,z\right)=I_{P0}\cdot \frac{w_{0x}}{w_{x}\left(z\right)}\frac{w_{0y}}{w_{y}\left(z\right)}\cdot e^{\frac{-2x^{2}}{w_{x}^{2}\left(z\right)}}\cdot e^{\frac{-2y^{2}}{w_{y}^{2}\left(z\right)}},\;w_{i}\left(z\right)=w_{0i}\sqrt{1+{\left(\frac{z}{z_{Ri}}\right)}^{2}}.$$ (4)

All five illumination geometries of **Fig 1** can be described, by choosing the independent beam-waist parameters *w*_*0x*_ and *w*_*0y*_ or Rayleigh ranges *z*_*Rx*_ and *z*_*Ry*_ accordingly. The generated local Raman signal and heat inside the sample are simultaneously proportional to the peak irradiance *I*_*P0*_. Considering a Gaussian detection pupil, *PSF*_*D*_ can be described as well by Eq 4. To meet the definition of a point spread function [28] the normalisation:

$$\int \int PSF_{D}\left(x,y,0\right)dxdy=1$$ (5)

is applied yielding to *PSF*_*D*_ units of m^-2^. For an ideal microscope, *PSF*_*D*_ is rotationally symmetric (*w*_*0x*_ = *w*_*0y*_ = *w*_*D*_) along the optical axis and exhibits a mirror-symmetry with respect to the focus plane. Thus, the point spread function of the detection optics in reflection and transmission geometry is:

$$PSF_{D}\left(x,y,z\right)=\frac{2}{\pi }\frac{1}{w_{D}^{2}\left(z\right)}\cdot e^{-2\frac{x^{2}+y^{2}}{w_{D}^{2}\left(z\right)}}$$ (6)

By identifying the coordinates of Eqs 4 and 6 with the ones in Eq 3 (consider additional coordinate breaks e.g. for light sheet and or in epi-illumination geometry), it is possible to derive the irradiance *I*_*D*_ at the detector for any microscope geometry. To get rid of experimental parameters in Eq 3, which are independent of the microscope, we introduce a signal factor *J*_*f*_ having units of a length:

$$I_{D}=I_{P0}\cdot \sigma_{R}\cdot N_{A}\cdot c\cdot \eta \cdot J_{f},\;J_{f}=\frac{1}{I_{P0}}\int \int \int I_{P}\left({\vec{r}}_{0}\right)\cdot PSF_{D}\left({\vec{r}}_{0}\right)d{\vec{r}}_{0}$$ (7)

In S1 Appendix, we describe the analytical derivation of the signal factors *J*_*f*_ for all five considered illumination geometries depicted in **Fig 1**.

### Light absorption insight the sample

The volumetric heat source *q*_*v*_(*r*,*t*), generated by linear absorption inside the sample at the position *r* is given by the illumination distribution *I*_*P*_(*r*,*t*) and the absorption coefficient *μ*_*a*_(*r*):

$$q_{V}\left(\vec{r},t\right)=\mu_{a}\left(\vec{r}\right)I_{P}\left(\vec{r},t\right)$$ (8)

For our comparison, the absorption coefficient *μ*_*a*_(*r*) is chosen to be spatially constant. Directly using the result of Gaussian optics (Eq 4) does of course neglect attenuation with penetration depth according to the Beer-Lambert law. The scattering coefficient *μ*_*s*_ of microscopical samples without dyes is often two orders of magnitude larger than the absorption coefficient (e.g. brain tissue near infrared: *μ*_*s*_ ≈ 10 mm^-1^, *μ*_*a*_ ≈ 0.1 mm^-1^ [28]). Due to the fact that imaging with ballistic light is anyway impossible after the first scattering length (*μ*_*s*_^*-1*^), the exponential decay caused by absorption can be neglected for discussing heat generation. We therefore employ an undepleted excitation approximation.

### Heat diffusion inside the sample

The heat equation [29,33–35] describes the evolution of temperature inside a sample depending on the volumetric heat source *q*_*v*_(*r*,*t*) being proportional to *I*_*P0*_. For a spatially and temporal constant thermal conductivity *k* (water 0.6 W·m^-1^·K^-1^), volumetric mass density *ϱ* (water 998 kg·m^-3^) and specific heat capacity *c*_*P*_ (water 4190 J·kg^-1^·K^-1^) the temperature distribution *u*(*r*,*t*) is given by:

$$\left[\frac{\partial }{\partial t}-\frac{k}{\varrho c_{p}}\nabla^{2}\right]u\left(\vec{r},t\right)=\frac{1}{\varrho c_{p}}q_{V}\left(\vec{r},t\right)$$ (9)

**Fig 3:**
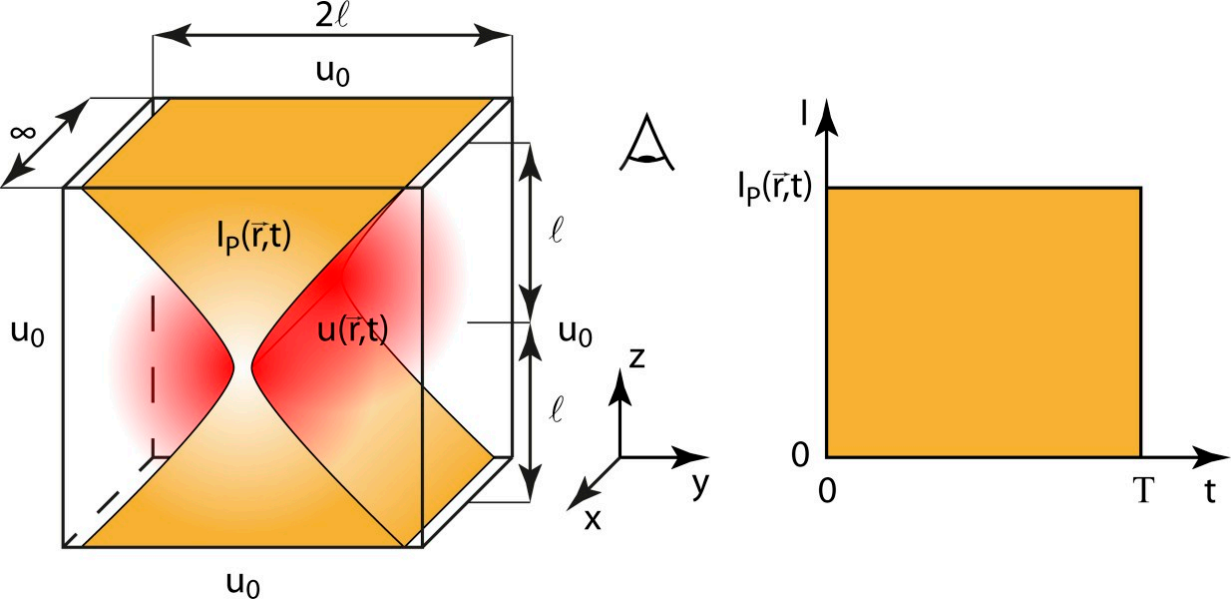
Boundary and source of the heat equation for line-confocal illumination. Left: cuboid sample (∞ × 2*l* × 2*l*) surrounded by a thermal reservoir of temperature *u*_*0*_ and typical illumination shape *I*_*P*_(*r*,*t*) of a line-confocal microscope. Right: evolution of irradiance with illumination period *T*.

In order to solve this equation for different illumination geometries (**Fig 1**), the geometry of the sample and the time evolution of the illumination need to be determined. We assume a cuboid sample with a characteristic length *l* describing the distance from the centre of illumination to the surrounding thermal reservoir, featuring a constant temperature *u*_*0*_ (**Fig 3** left). To simplify the integration and to avoid edge effects, the symmetry of the cuboid sample is adapted to the illumination: 2*l* × 2*l* × ∞ for line-confocal and line illumination and 2*l* × ∞ × ∞ for light sheet illumination. The time dependent illumination *I*_*P*_(*r*,*t*) is defined to be constant during a period *T* and zero otherwise (**Fig 3** right):

$$\begin{array}{l} I_{P}\left(\vec{r},t\right)=\{\begin{array}{ll} \;\;\;0, & \;t\in \left(-\infty ,0\right)\\ I_{P}\left(\vec{r}\right), & \;t\in \left[0,T\right]\\ \;\;\;0, & t\in \left(T,\infty \right) \end{array} \end{array}$$ (10)

As a further simplification we assume a constant start temperature distribution (scanning is discussed below):

$$u\left(\vec{r},0\right)=u_{0}$$ (11)

To solve the heat equation (Eq 9) for a wide range of illumination periods *T* (very short illumination period: no heat diffusion, short period: heat diffusion while not reaching the thermal reservoir, long period: heat diffusion reaching the thermal reservoir, thermally stationary state: Laplace's equation) we employ different analytical and numerical solution strategies (**Table 1**). In S2 Appendix, we describe the analytical derivation for the diffusion free case. The waist diameters for the elliptic Gaussian illumination beam are chosen according to **Table 2**. For choosing the heat conduction parameter the sample is assumed to consist mainly of water and the illumination wavelength *λ* is set to 550 nm. The calculations are performed for three different characteristic lengths *l* (10 μm, 100 μm, 1000 μm) of the sample being relevant in case heat diffusion reaches the thermal reservoir.

**Table 1:** Strategies to solve the heat equation for a wide range of illumination periods *T* and different illumination geometries.

Illumination geometry	Diffusion without reaching the reservoir	Diffusion reaching the reservoir in *T*
**Point confocal**	Numerical convolution (3D heat kernel)	Not calculated (*T* is usually small)
**Line-confocal**	Numerical convolution (2D heat kernel)	2D Finite element method (FEM)
**Light sheet**	Analytical convolution (1D heat kernel)	1D Finite element method (FEM)
**Light line**	Analytical convolution (2D heat kernel)	2D Finite element method (FEM)
**Wide-field**	Analytical (diffusion free)	Not calculated (infinite sample)

**Table 2:** Waist diameter of the elliptic Gaussian beam defining the illumination geometry *I*_*P*_(*r*).

Illumination geometry	*w*_*P0x*_	*w*_*P0y*_	*w*_*P0z*_
**Point confocal**	0.8 NA → 175 nm	0.8 NA → 175 nm	propagation direction
**Line-confocal**	→ ∞	0.8 NA → 175 nm	propagation direction
**Light sheet illumination**	propagation direction	→ ∞	*w*_*Pz*_(*x*) = 2.5 μm
**Line illumination**	propagation direction	*w*_*Py*_(*x*) = 2.5 μm	*w*_*Pz*_(*x*) = 2.5 μm
**Wide-field illumination**	→ ∞	→ ∞	propagation direction

**Fig 4:**
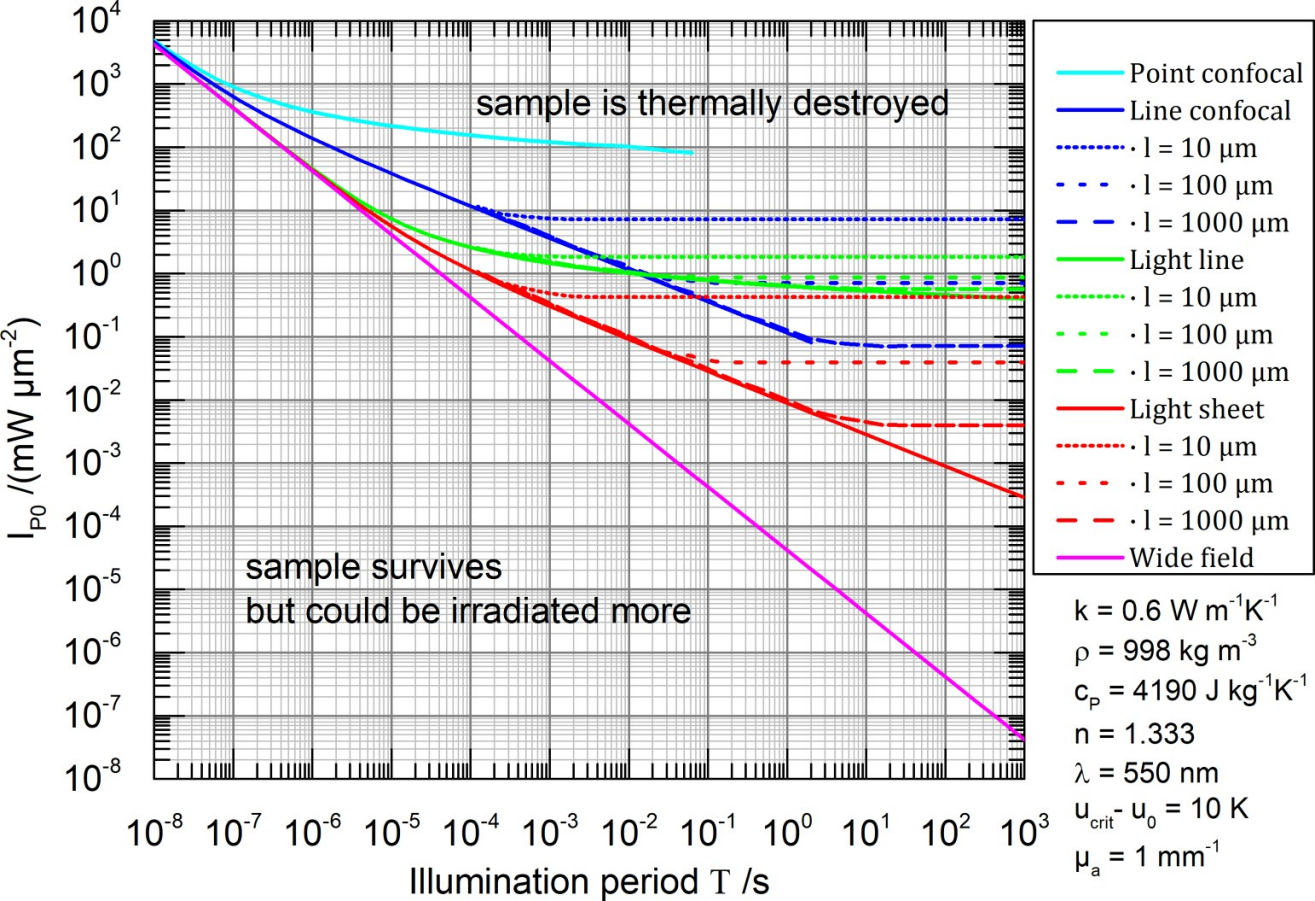
Maximal permissible peak irradiance *I*_*P0*_(*T*) for five different illumination geometries depending parametrically on the illumination period *T* using a 0.8 NA lens. The sample is assumed to consist mainly of water. The vacuum illumination wavelength is chosen to be *λ* = 550 nm. The maximal permissible temperature rise is arbitrarily set to *u*_*crit*_ *- u*_*0*_ = 10 K and the absorption coefficient is set to *μ*_*a*_ = 1 mm^-1^. Solid lines describe infinitely extended samples (heat never reaches the thermal reservoir) and dashed lines describe samples of a finite size with a cuboid shape: 2*l* × 2*l* × ∞ for line-confocal and line illumination and 2*l* × ∞ × ∞ for light sheet illumination. Results of the thermally stationary state are calculated with FEM and match the analytical solution.

The solutions of the heat equation *u*(*r*,*T*) are variations in temperature, depending linearly on the peak irradiance *I*_*P0*_ of the illumination field (see Eqs 9, 8 and 5). However, only the maximal temperature reached in the sample (expected at the end of illumination period *T*) is of interest to us being linear in *I*_*P0*_, too. In general the solutions *u*(*r*,*T*) of the heat equation can further be solved for the maximal permissible peak irradiance *I*_*P0*_(*T*) depending on the maximal permissible temperature rise (*u*_*crit*_ *- u*_*0*_) and parametrically on *T*. The maximal permissible peak irradiances *I*_*P0*_(*T*) are calculated for the five different illumination geometries leading to the results presented in **Fig 4**.

Each graph in **Fig 4** can be understood as slicing the parameter space in two halves. In the upper half, the sample is thermally destroyed and in the lower half the sample survives but could be irradiated more. The *I*_*P0*_(*T*) graph itself determines for a selected illumination geometry the most efficient irradiance.

The very local excitation of point confocal microscopy features the best properties in terms of cooling, leading to the least irradiance constraints. Line-confocal microscopy features higher parallelization but especially thick samples are badly cooled by the thermal reservoir. A light sheet illumination enables by far the highest parallelization, but suffers from a rather bad cooling, requiring an irradiance attenuation of multiple orders of magnitude. An extreme case is given by wide-field illumination of a large sample. It particularly shows no cooling effect whatsoever. The main finding so far is: The desirable acceleration of imaging through parallelization is counteracted by a heat build-up in the sample. For thick samples, parallelization is thermally contra productive.

Thermal interaction between sequential scan positions can be neglected in case the maximal permissible irradiance *I*_*P0*_(*T*) is constant with respect to the Illumination period *T* (thermally stationary states: horizontal graphs in **Fig 4**). In other words: when scanning is slower than thermal diffusion, holding an illumination position allows for the same irradiance. This applies especially for realistic scan times of the point confocal microscope and the light line illumination (see Discussion section: high scan speed, here < 1 ms per pixel or line). Light sheet and wide-field illumination are anyway non scanning techniques, so the interaction question does not apply (light sheet Raman z-scans are slow compared to thermal diffusion [36]). The only problematic case in terms of thermal interaction between scan positions is line-confocal microscopy where thick samples are scanned fast enough to not reach the thermally stationary state. In this case *I*_*P0*_(*T*) might be estimated optimistically too high. Structured illumination microscopy (SIM) [37], for acquisition of spatial frequencies outside the optical transfer function, shows the same thermal properties as wide-field microscopy.

### Temperature factors of different illumination geometries

**Fig 5:**
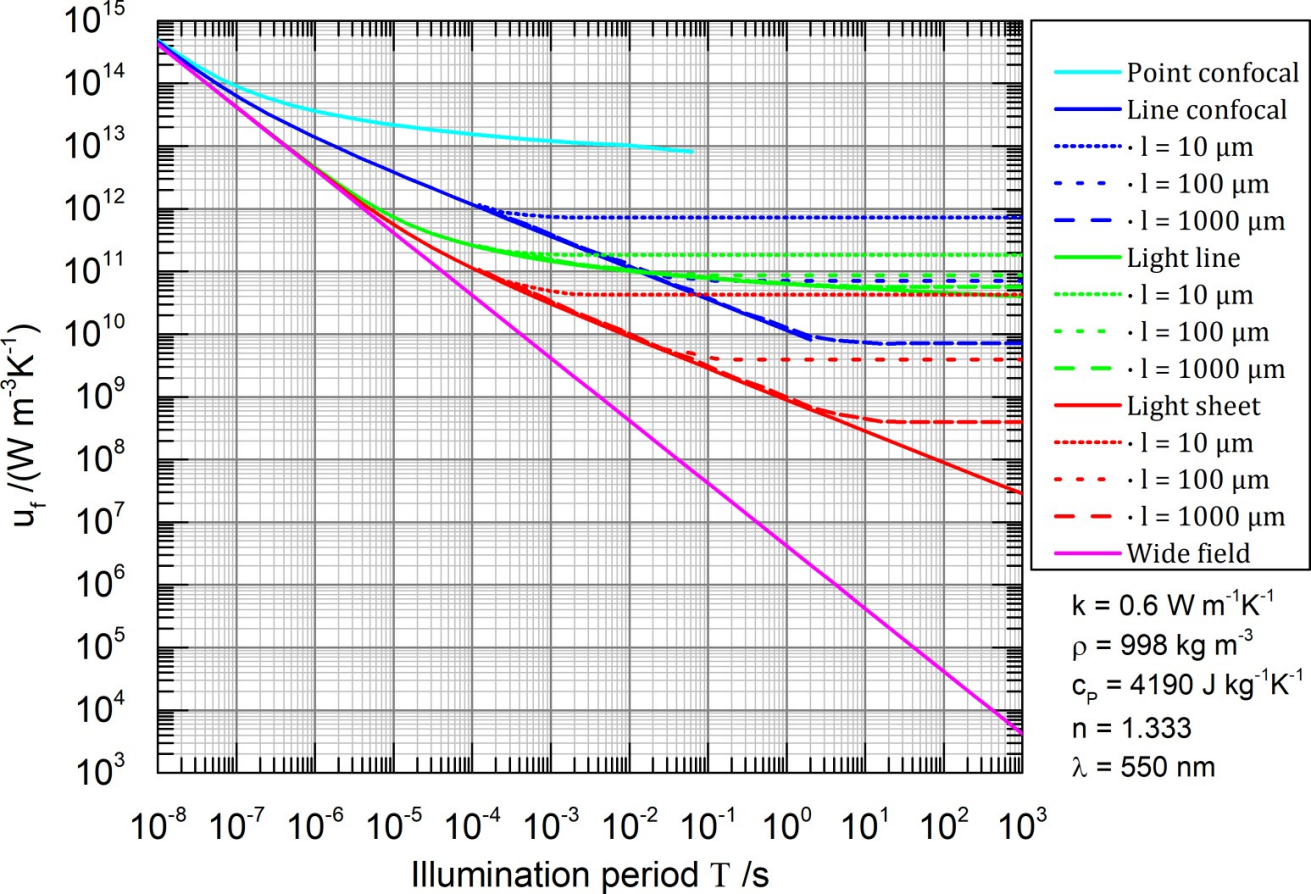
Temperature factor *u*_*f*_(*T*) for five different illumination geometries depending parametrically on the illumination period *T* using a 0.8 NA lens. Like **Fig 4** but normalised against the absorption coefficient *μ*_*a*_ and the maximal permissible temperature rise (*u*_*crit*_ *- u*_*0*_).

Due to linearity the solutions of *I*_*P0*_(*T*) can all be normalised for the absorption coefficient *μ*_*a*_ and the maximal permissible temperature rise (*u*_*crit*_ *- u*_*0*_). For this we introduce a comparable temperature factor *u*_*f*_(*T*) only depending on the illumination geometry, the illumination period *T* and the least possible number of material constants:

$$I_{P0}\left(T\right)\leq \frac{\left(u_{crit}-u_{0}\right)}{\mu_{a}}\cdot u_{f}\left(T\right)$$ (12)

where for example in case of no diffusion (see wide-field graph **Fig** 5 and S2 Appendix) *u*_*f*_(*T*) is:

$$u_{f}\left(T\right)=\frac{\varrho c_{p}}{T}$$ (13)

The temperature factors for all five illumination geometries are presented in **Fig** 5. The actual values of the absorption coefficient *μ*_*a*_ and temperature threshold *u*_*crit*_ are not decisive for the comparison of different illumination geometries. Essential is only the existence of a given permissible temperature threshold *u*_*crit*_.

### Signal-to-noise ratio of light microscopes

The *SNR* of an image is defined as the ratio of signal *S* and the standard deviation of the noise *N*. The noise can consist of different, independent sources. We discuss the Poisson-distributed shot noise *N*_*p*_ of the signal photons *S*, the shot noise *N*_*b*_ of additional out-of-focus or background light S_b_ and an uncorrelated detector noise *N*_*d*_:

$$SNR=\frac{S}{N}=\frac{S}{\sqrt{N_{p}^{2}+N_{b}^{2}+N_{d}^{2}}}=\frac{S}{\sqrt{S+S_{b}+N_{d}^{2}}}$$ (14)

It is convenient to introduce a noise condition parameter *q*_*s*_:

$$SNR=\frac{1}{\sqrt{1+q_{S}^{2}}}\cdot \sqrt{S}\;were\;q_{S}^{2}=\frac{N_{b}^{2}+N_{d}^{2}}{N_{p}^{2}}=\frac{S_{b}+N_{d}^{2}}{S}$$ (15)

In case of an ideal detection, *q*_*s*_ = 0 describes only the shot noise of signal photons, while *q*_*s*_ = 1 indicates an equal amount of additional noise produced by out-of-focus light and for detector readout noise.

The detected signal *S* of every pixel is proportional to the irradiance *I*_*D*_ (Eq 7) at the detector, the apparent pixel size *A* and the illumination period *T*:

$$S=I_{D}\cdot A\cdot T$$ (16)

Depending on the chosen image scan technique (data acquisition strategy), the illumination period *T* is given by the total 2D image acquisition time *T*_*0*_, the number of pixels in x-direction *n*_*x*_ and the y-direction *n*_*y*_ according to **Table 3**.

**Table 3:** Image scanning types. The illumination period *T* is given by the total 2D image acquisition time *T*_*0*_.

Illumination geometry	Associated spatial scan technique	Illumination period *T*
**Point confocal**	0D (whisk broom)	*T*_*0*_* · n*_*x*_^*-1*^* · n*_*y*_^*-1*^
**Line-confocal**	1D (push broom)	*T*_*0*_* · n*_*y*_^*-1*^
**Light sheet illumination**	2D (framing)	*T*_*0*_
**Line illumination**	1D (push broom)	*T*_*0*_* · n*_*y*_^*-1*^
**Wide-field illumination**	2D (framing)	*T*_*0*_

The best achievable *SNR* of an image with temperature limited irradiance is given by Eqs 15, 16, 7 and 12:

$$SNR=\frac{1}{\sqrt{1+q_{S}^{2}}}\cdot \sqrt{\frac{\left(u_{crit}-u_{0}\right)}{\mu_{a}}}\cdot \sqrt{u_{f}\left(T\right)}\cdot \sqrt{\sigma_{R}\cdot N_{A}\cdot c\cdot A}\cdot \sqrt{J_{f}}\cdot \sqrt{T}$$ (17)

For a comparison between various acquisition strategies it is convenient to normalise against experimental parameters which are independent of the microscopy system leading to:

$$SNR_{N}=\frac{1}{\sqrt{1+q_{S}^{2}}}\cdot \sqrt{u_{f}\left(T\right)\cdot \eta \cdot J_{f}\cdot T}$$ (18)

The absolute *SNR* is thus given by the normalised *SNR*_*N*_:

$$SNR=SNR_{N}\cdot \sqrt{\frac{\left(u_{crit}-u_{0}\right)}{\mu_{a}}}\cdot \sqrt{\sigma_{R}\cdot N_{A}\cdot c\cdot A}$$ (19)

### Example: *SNR* of a wide-field microscope

For idealised wide-field illumination, heat transfer can be neglected as the whole volume is heated homogeneously, leading to the temperature factor *u*_*f*_(*T*) given by Eq 13. The signal factor *J*_*f*_ for a wide-field microscope can be easily calculated from Eq 3 leading to *J*_*f*_ = 2*l* (see S1 Appendix). Because wide-field microscopy does not feature optical sectioning, the collected light depends under the undepleted excitation approximation just on the sample thickness. However, light which is collected out-of-focus, does not contribute to a sharp image. We therefore consider the in-focus signal factor to correspond to the depth of focus [38]:

$$J_{f}=2\frac{\lambda n}{{\text{NA}}^{2}}$$ (20)

To account for the additional shot noise produced by the out-of-focus light the noise condition parameter *q*_*s*_ is used. Neglecting dark noise of the detector, *q*_*s*_^2^ can be identified with the ratio of out-of-focus light and in-focus light using Eqs 15, 16 and 7 (assuming 2*l* ≥ *J*_*f*_):

$$q_{S}^{2}=\frac{2l-J_{f}}{J_{f}}=\frac{2l}{J_{f}}-1$$ (21)

Independent of the illumination period *T*, the normalised *SNR* for a wide-field microscope using Eqs 18, 13, 20 and 21 becomes:

$$SNR_{N}=\frac{1}{\sqrt{2l}}\cdot \sqrt{\varrho c_{p}}\cdot \frac{2\eta \lambda n}{NA^{2}}$$ (22)

The constant behaviour according to the illumination period *T* is caused by the absence of a heat dissipating mechanism. Under these idealised conditions, a longer exposure does automatically require a reduced irradiance (**Fig** 5) in a way that the *SNR* cannot be improved. The normalised *SNR* can be calculated similarly for any other type of microscope.

## Results

The developed microscope model is used to compare the achievable *SNR* of the five different illumination geometries common for light microscopy (**Fig 1**). In order to make the comparison independent of as many experimental parameters as possible, we have introduced the concept of the normalized *SNR*_*N*_ (Eq 19). A constrained image acquisition time *T*_*0*_ and a fixed permissible temperature rise *u*_*crit*_ *- u*_*0*_ being safe for the sample are assumed. To always meet this temperature constraint, the irradiance *I*_*P0*_ is adjusted parametrically depending on image acquisition time *T*_*0*_.

**Fig 6:**
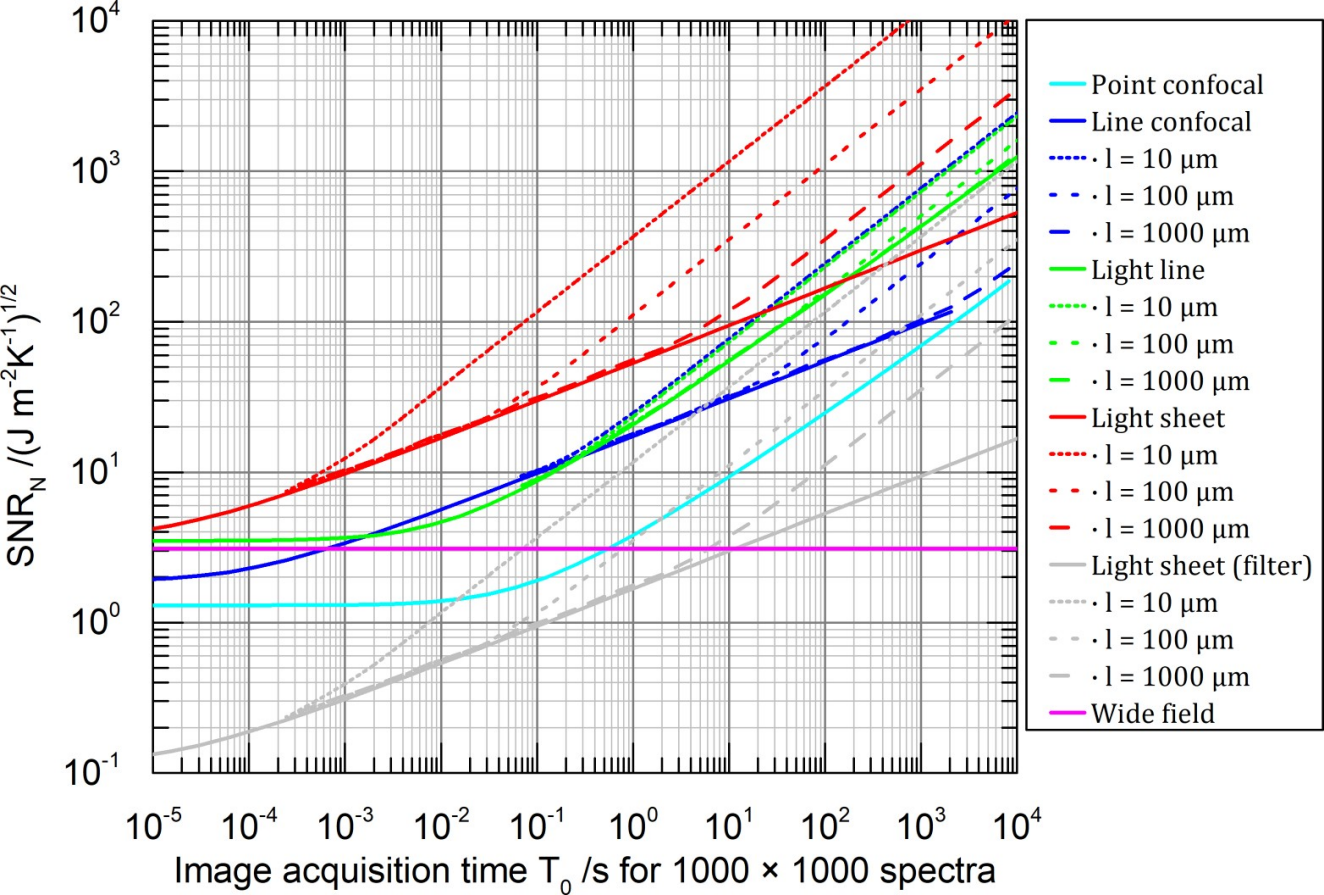
Theoretical *SNR*_*N*_ comparison of aqueous sample images consisting of 1000 × 1000 spectra, using a 0.8 NA objective lens. We assume an illumination wavelength of *λ* = 550 nm, temperature limited irradiance, ideal hyperspectral sensors (except grey line): efficiency *η* = 1 and a noise condition parameter *q*_*s*_ = 0. Solid lines describe infinitely extended samples (heat never reaches the thermal reservoir) and dashed lines describe samples of a finite size with cuboid shape: 2*l* × 2*l* × ∞ for line-confocal and line illumination and 2*l* × ∞ × ∞ for light sheet illumination. The grey curve represents a light sheet system equipped with a 1000 channel filter based hyperspectral detector. Assuming a constant spectral light distribution and shot noise only the SNR drops by a factor of square root of 1000 compared to the ideal hyperspectral detector (red curve). Notice: the irradiance is adjusted parametrically with acquisition time *T*_*0*_ according to **Fig** 5, to always meet the permissible temperature rise in the sample.

**Fig 7:**
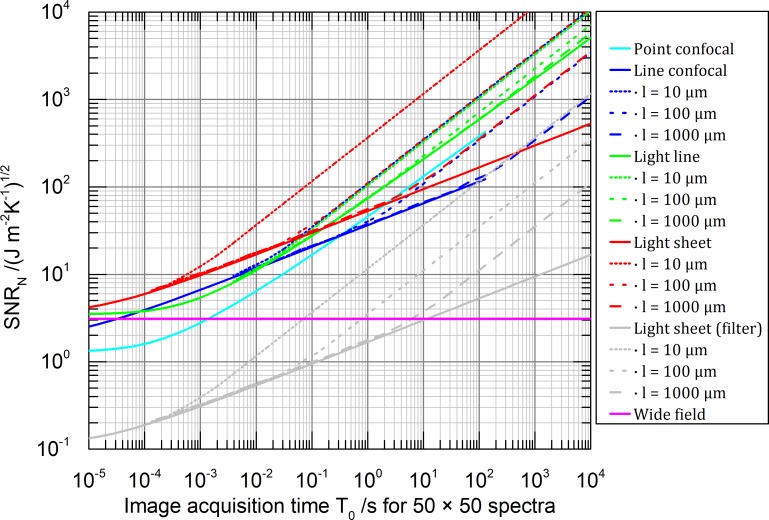
Like Fig 6 but for 50 × 50 spectra. Notice, that for a shrinking number of pixels (accompanied by a shrinking field of view), all methods (apart from grey) exhibit similar performance (graphs shift along *T*_*0*_) because they become essentially more and more scanning approaches.

The results for images of aqueous 3D volume samples, illuminated with 550 nm and observed with a 0.8 NA objective lens (**Table 2**) consisting of 1000 × 1000 and 50 × 50 pixels are shown in **Figs 6** and **7** respectively. For all microscope types we assume the noise condition parameter *q*_*s*_ = 0 essentially neglecting detector noise and neglecting additional shot noise produced by background light. Background light can be neglected for microscope types featuring intrinsically optical sectioning (confocal types, light line and light sheet). The detection efficiency is assumed to be (*η* = 1). For other values of *η* the *SNR*_*N*_ would change by a constant factor of the square rood of *η*. The corresponding temperature factors, describing the accounted irradiance adjustment of *I*_*P0*_ (Eq 12) are given in **Fig** 5.

A user of a point-confocal Raman microscope can read the graph as follows: Let us assume 100 s are usually enough to get a decent hyperspectral, confocal Raman image consisting of 50 × 50 spectra (40 ms for each spectrum, 2 s for each line) of a particular sample with a chosen permissible laser power. This leads according to **Fig 7** to a *SNR*_*N*_ ≈ 350. Observing for example the graph for the line-confocal microscope at the same *SNR*_*N*_ level, this instrument can produce the same image quality in terms of *SNR* within 10 s exposure in case the sample has a thickness of 2 × 10 μm and it is further surrounded by a thermal bath. With a 10 times thicker sample however, we can expect only the same imaging speed as the point-confocal technique. This result seams counterintuitive because parallelization does not positively affect here the imaging speed. But one should notice, to maintain the same maximum temperature insight the sample like in the point confocal microscope, the irradiance has to be attenuated for the line-confocal microscope by one (for 2 × 10 μm) respectively two (for 2 × 100 **μ**m) orders of magnitude (see **Figs 4** and **5**, point confocal at 40 ms, line-confocal at 2 s).

For short image acquisition time (*T*_*0*_ = 10^−5^ s) the graphs of light line (green), light sheet (red) and wide-field microscopy (magenta) in **Figs 6** and **7** are roughly at equal *SNR*_*N*_ stemming from the converging temperature factors *u*_*f*_(*T*) (**Fig** 5 for *T* < 10^−5^ s). The deviation of the confocal techniques does not originate from the temperature factors but from the assumed delta like detection pinhole which is an idealized simplification. A real pinhole or slit can reduce the *SNR*_*N*_ even further [1,30,31].

The graphs in **Figs 6** and **7** show three different slopes for large acquisition times:

1. *SNR*_*N*_ = *const*.: No heat diffusion. Achievable image quality is independent of the image acquisition time *T*_*0*_ (wide-field microscope)
2. ${SNR}_{N}\propto \sqrt[{4}]{T_{0}}$: One dimensional heat diffusion. (light sheet- and line-confocal illumination)
3. ${SNR}_{N}\propto \sqrt{T_{0}}$: Two-dimensional heat diffusion (point confocal- and line illumination) or thermally stationary state, were a finite sized sample is cooled after long time completely by its surface touching the thermal reservoir.

So far, we did not discuss the need of hyperspectral sensing and data acquisition for Raman microscopy. We always assumed an ideal hyperspectral 0D, 1D or 2D detector being able to sort every incoming photon according to its wavelength and origin without loss or additional encoding noise. Fortunately, there are instruments available (typically based on dispersive elements such as prisms or gratings) that are almost ideal. For 2D imaging they are referred to as integral field spectrographs [39,40]. Note: integral field spectrographs are intrinsically snapshot hyperspectral imager but not all snapshot hyperspectral imager are free from loss or additional encoding noise.

However, for images containing more than 1000 pixel it is technically challenging to record the whole hyperspectral information simultaneously. For more spectra it is necessary to employ techniques, which scan at least one spatial or spectral dimension sequentially. In case of Raman microscopy, spatial scanning is related to a limited field-of-illumination which can be beneficial in terms of cooling allowing for increased irradiance. Spectral scanning is related to discarding photons by filtering methods or adding encoding noise e.g. by Fourier transform spectroscopy [41] which is of course disadvantageous but enables a large simultaneous field of view.

According to the chosen imaging spectrometer, the *SNR*_*N*_ can drop by an additional factor. For example, considering shot noise only, a wide-field- or light sheet illumination combined with a tuneable narrow bandpass for the detection of 1000 independent spectral channels such as a Lyot- or tuneable Bragg-filter will decrease on average the *SNR* by square root of 1000 (grey graph **Figs 6** and **7**). A detailed *SNR* discussion of different imaging spectrometer classes can be found in [42,43].

## Discussion

The results presented in **Figs 6** and **7** can form the basis for developing the fastest Raman microscope technique. Consider a confocal Raman microscope equipped with an efficient grating spectrometer taking 2300 s for 1000000 spectra to gain a *SNR*_*N*_ = 100—which might turn out to be sufficient for the chosen 3D sample (cyan in **Fig 6**). A light sheet microscope equipped with an ideal hyperspectral imaging detector is able to produce a comparable image of a 2 × 10 μm thin sample in 0.08 s and is thus more than 10000× faster (red dotted in **Fig 6**). For a thicker, less efficiently cooled 3D sample however, the irradiance has to be reduced (**Fig** 5) and so the benefit of parallel illumination drops easily by a factor of 100 (red long dash in **Fig 6**). Since no technical solution to measure 1000000 spectra simultaneously actually exists, either the field of view has to be reduced (**Fig 7**) or a filtering technique based on Lyot- or tuneable Bragg-filter could be combined with the light sheet technique instead. The loss of signal accompanied with filtering however results in a drop of *SNR* (grey in **Fig 6**) which can thus be beaten by a point-confocal Raman microscope. Therefore, a light sheet microscope equipped with an integral field spectrograph turns out to be the best choice only for a thin sample and a reduced field of view (**Fig 7**). Notice that, for a decreasing number of spectra, all methods exhibit similar performance because they become essentially more and more scanning approaches.

An interesting solution for highly transparent 3D samples is the light-line illumination in combination with a 1D (push broom) grating-based spectrometer equipped with a slit. The limited illumination, the efficient cooling of the thin light beam configuration and the spectral encoding optimised for shot noise promise a 60× speedup in comparison to the confocal technique even for thick samples (green in **Fig 6**).

Until now we discussed 3D samples with 3D distributed heat sources. To investigate the surface of non-transparent 2D samples with Raman, only wide-field and confocal techniques are practically applicable. In the case of diffusion-limited surface cooling, the effect of a wide-field illumination on a non-transparent sample is equivalently described by a thin sample being illuminated by a homogeneous, undepleted light sheet (red dotted in **Figs 6** and **7**). Comparing light sheet and both confocal techniques (red, cyan, blue dotted in **Figs 6** and **7**), the speed scales roughly linear with their parallelization due to the reduced thermal diffusion possibilities in 2D. We conclude that only for efficiently cooled 2D samples the claim of linear speed improvement in line-scan Raman microscopy [10] holds true. The same applies to multi confocal techniques [12]. For thick samples and long scan lines however, the line-confocal microscope can be left behind by the point confocal method in terms of working speed (cyan, blue in **Fig 7**). For multi confocal techniques and thick samples, the working speed depends on the exact shape of the illumination pattern. In case the spacing between the illumination PSFs enables thermal interaction, the cooling and so the maximal permissible irradiance can decrease like for line-confocal or even wide-field techniques.

We found that thermal interaction between sequential scan positions can be neglected for determining the maximal permissible irradiance *I*_*P0*_(*T*) in cases where scanning is slower than thermal diffusion (horizontal graphs **Fig 4**). In cases where scanning is faster than thermal diffusion, one illumination technique turns thermally into the next, more parallel one (point confocal → line-confocal → wide-field, light line → light sheet). For instance, a point confocal microscope which is scanning a single line very quickly or even multiple times behaves thermally like a line-confocal microscope.

Technically, the line sensor readout speed of a point-scanning spectrometer may be limited, which is why the sampling frequency of a confocal microscope remains limited. However, the multiple scanning of a line can be solved purely optically with a scan-descan-rescan approach as it is described in [13]. A 2D image sensor can be used, which is read out only once after every multiple scanned line. This way, the sectioning advantage of the isotropic confocal microscope is combined with the thermal advantage of a line-confocal one. Notice that a significant increase in irradiance above typical confocal Raman microscopes (300 mW·μm^-2^) [36] can cause an avalanche breakdown to become a relevant destruction mechanism (10000 mW·μm^-2^) [44].

It should be noted that all results reflect the behaviour of idealized systems. In praxis there will be no strict separation between the sample and the thermal reservoir. Inhomogeneous properties of a real sample can lead to deviant behaviour in practical measurements. Nevertheless, our results help to understand the speed limitations in Raman microscopy and pave the way for future development in the field. The first strength of our microscope model is the ability to exclude experimental parameters, which might be anyway hard to access in a practical comparison of microscopy systems. Secondly, the presented microscope model grants the flexibility to assess microscopy systems, which are not even developed yet.

Before performing these theoretical investigations, we managed to speed up 3D Raman microscopy by using a combination of light sheet illumination and imaging Fourier transform spectroscopy [36]. We gained an experimental speed improvement of a factor 5.3 in comparison to the confocal microscope imaging of a zebrafish embryo up to a depth of 150 μm. The conditions are comparable with the grey graph in **Fig 6** where the *SNR*_*N*_ = 100. For a 200 μm thick sample, a 2.9 times speed improvement and for a sample of 20 μm thickness a 31 times speed improvement is predicted. This experimental result proves the resilience of our model, despite all approximations and assumptions.

Our model can be applied beyond Raman applications to other types of microscopy and micro-spectroscopy where absorption is crucial and operating speed is limited by thermal effects. Even though the significantly higher effective cross section of fluorescence permits lower irradiances for the same frame rates, the requirements placed on fluorescence microscopy are increased to the same extent. Fluorescence volumetric imaging at video rate can also suffer from thermal issues in living organisms.

## Supporting information

S1 Appendix Calculating the signal factor *J*_*f*_. (DOCX)

S2 Appendix Calculating *u*_*f*_(*T*) in case of no diffusion. (DOCX)
