# Supplementary material for: Thermal illumination limits in 3D Raman microscopy: A comparison of different sample illumination strategies to obtain maximum imaging speed
Source: PLoS One. 2019 Aug 13;14(8):e0220824. doi: 10.1371/journal.pone.0220824 (PMC6692011; doi:10.1371/journal.pone.0220824)
Supplement: S1 Appendix — (DOCX) [file pone.0220824.s001.docx]

Calculating the signal factor *J_f_* (S1)

The signal factor *J_f_* is calculated for all five illumination geometries namely: point- and line-confocal microscopes as well as light line-, light sheet- and wide-field illumination. According to equation 7 the signal factor *J_f_* is defined as:

$$I_{D}=I_{P0}\cdot\sigma_{R}\cdot N_{A}\cdot c\cdot\eta\cdot J_{f}, J_{f}=\frac{1}{I_{P0}}\iiint I_{P}\left( \vec{r}_{0} \right)\cdot{PSF}_{D}\left( \vec{r}_{0} \right)d\vec{r}_{0}$$

. (7)

The irradiance distribution of an elliptic Gaussian beam with propagation in z-direction is given by equation 4:

$$I_{P}\left( x,y,z \right)=I_{P0}\cdot\frac{w_{0x}}{w_{x}\left( z \right)}\frac{w_{0y}}{w_{y}\left( z \right)}\cdot e^{\frac{-2x^{2}}{w_{x}^{2}\left( z \right)}}\cdot e^{\frac{-2y^{2}}{w_{y}^{2}\left( z \right)}}, w_{i}\left( z \right)=w_{0i}\sqrt{1+\left( \frac{z}{z_{Ri}} \right)^{2}}$$

. (4)

By choosing the two independent beam-waist parameters *w_0x_* and *w_0y_* and the peak irradiance *I_P0_*, it is possible to describe all five illumination geometries.

For an ideal microscope with Gaussian detection pupil, the point spread function of the detection optics in reflection and transmission geometry is given by equation 6:

$${PSF}_{D}\left( x,y,z \right)=\frac{2}{\pi}\frac{1}{w_{D}^{2}\left( z \right)}\cdot e^{-2\frac{x^{2}+y^{2}}{w_{D}^{2}\left( z \right)}}$$

. (6)

By identifying the coordinates of equations 4 and 6 with the ones in equation 7 (consider coordinate breaks e.g. for light sheet and or in epi-illumination geometry), the signal factor *J_f_* for the five geometries is in the following calculated analytically.

# 1. Point-confocal microscope

The illumination distribution *I_P_* and the detection point spread function of an ideal point-confocal microscope are featuring rotational symmetry along the optical axis (z-direction) and mirror symmetry along the xy-plane. The Gaussian beam parameter of equation 4 are thus given by: *w_0x_* = *w_0y_* = *w_0_*. The illumination distribution *I_P_* with propagation in z-direction is thus given by:

$$I_{P}\left( x,y,z \right)=I_{P0}\cdot\frac{w_{P0}^{2}}{w_{P}^{2}\left( z \right)}\cdot e^{-2\frac{x^{2}+y^{2}}{w_{P}^{2}\left( z \right)}}$$

. (A1)

The Stokes shift (Raman: ≤ 3500 cm^-1^) can be neglected, so that in case of equal or identical optical systems for illumination and detection the beam-waist parameter are set:

$$w_{P}\left( z \right)=w_{D}\left( z \right)=w\left( z \right)$$

. (A2)

The signal factor (equation 7) calculates using the detection optics point spread function (equation 6) and the illumination distribution (equation A1) both with optical axis in z‑direction:

$$J_{f}=\frac{2}{\pi}\iiint\frac{w_{0}^{2}}{w^{4}\left( z \right)}\cdot e^{-4\frac{x^{2}+y^{2}}{w^{2}\left( z \right)}}dxdydz$$

, (A3)

$$J_{f}=w_{0}^{2}\frac{n\pi^{2}}{2\lambda}$$

. (A4)

Expressing the divergence of the Gaussian beam (half opening angle θ) by the numerical aperture $NA=n\sin\theta$ leads to:

$$w_{0}^{2}=\lambda^{2}\frac{n^{2}-{NA}^{2}}{n^{2}\pi^{2}\cdot{NA}^{2}}$$

, (A5)

and yields:

$$J_{f}=\frac{\lambda}{2}\cdot\frac{n^{2}-{NA}^{2}}{n\cdot{NA}^{2}}$$

. (A6)

For comparison, the axial resolution of a confocal microscope is given by [1]:

$$d_{z}=\frac{n\lambda}{2\cdot{NA}^{2}}$$

. (A7)

# 2. Line-confocal microscope

In contrast to the point-confocal microscope, the illumination of a line-confocal microscope is extended in one direction (w.l.o.g. in x-direction). Practically this extension can be implemented in different ways: By expanding the radiation of a spatially coherent light source in one direction, by imaging an incoherent line shaped light source or by scanning of a point light source. For the transparent and incoherently emitting sample assumed here, these illuminations can be considered as equivalent. It is therefore convenient to describe the illumination in y-direction Gaussian shaped and in x-direction collimated ($w_{0x}\to\infty$):

$$I_{P}\left( x,y,z \right)=I_{P0}\cdot\frac{w_{P0y}}{w_{Py}\left( z \right)}\cdot e^{-2\left( \frac{y}{w_{Py}\left( z \right)} \right)^{2}}$$

. (A8)

The Stokes shift (Raman: ≤ 3500 cm^-1^) can be neglected, so that in case of equal or identical optical systems for illumination and detection the beam-waist parameters are set:

$$w_{Py}\left( z \right)=w_{D}\left( z \right)=w\left( z \right)$$

. (A9)

The signal factor (equation 7) using the detection optics point spread function (equation 6) and the illumination distribution (equation A8) both with optical axis in z-direction calculates to:

$$J_{f}=\iiint\frac{w_{0}}{w\left( z \right)}\cdot e^{-2\frac{y^{2}}{w^{2}\left( z \right)}}\cdot\frac{2}{\pi}\frac{1}{w^{2}\left( z \right)}\cdot e^{-2\frac{x^{2}+y^{2}}{w^{2}\left( z \right)}}dxdydz$$

. (A10)

Since this expression is divergent, it leads to a finite result only for samples of finite height ∆*z*:

$$J_{f}=\frac{2}{\pi}\int_{-\frac{\Delta z}{2}}^{\frac{\Delta z}{2}} \iint\frac{w_{0}}{w^{3}\left( z \right)}\cdot e^{-2\frac{x^{2}+{2y}^{2}}{w^{2}\left( z \right)}}dxdydz$$

, (A11)

$$J_{f}=\frac{\sqrt{2}n\pi}{\lambda}w_{0}^{2}\cdot\log\left[ \frac{\Delta z\cdot\lambda+\sqrt{{\Delta z}^{2}\lambda^{2}+4n^{2}\pi^{2}w_{0}^{4}}}{2n\pi w_{0}^{2}} \right]$$

. (A12)

By choosing ∆*z*, it is possible to distinguish between light scattered throughout the whole sample and that, scattered in a thin layer around the focus only.

# 3. Light line illumination

Connecting a line scanning microscope with an illumination directed orthogonal to the direction of detection, results in a collimated light line, so that only the in focus part of the sample is illuminated. In practice, such a collimated beam can be generated from a weakly focused, round Gaussian beam. To exclude the compromise between diameter and divergence from the discussion, we approximate the illumination line by a collimated beam with 2 · *w_P0_* diameter. The intensity distribution of such a light line (w.l.o.g. in x-direction) can be described by:

$$I_{P}\left( x,y,z \right)=I_{P0}\cdot e^{-2\frac{y^{2}+z^{2}}{w_{P0}^{2}}}$$

. (A13)

The signal factor (equation 7) using the detection optics point spread function (equation 6) with an optical axis in z-direction and the illumination distribution (equation A13) with an optical axis in x-direction calculates to:

$$J_{f}=\iiint e^{-2\frac{y^{2}+z^{2}}{w_{P0}^{2}}}\cdot\frac{2}{\pi}\frac{1}{w_{D}^{2}\left( z \right)}\cdot e^{-2\frac{x^{2}+y^{2}}{w_{D}^{2}\left( z \right)}}dxdydz$$

, (A14)

$$J_{f}=\frac{n\pi\cdot w_{P0}\cdot w_{D0}}{\lambda}\cdot e^{\frac{{n^{2}\pi}^{2}\cdot w_{D0}^{2}\cdot\left( w_{P0}^{2}+w_{D0}^{2} \right)}{w_{P0}^{2}\cdot\lambda^{2}}}\cdot K_{0}\left( \frac{{n^{2}\pi}^{2}\cdot w_{D0}^{2}\cdot\left( w_{P0}^{2}+w_{D0}^{2} \right)}{w_{P0}^{2}\cdot\lambda^{2}} \right)$$

. (A15)

Were *K_n_*(*z*) is the modified Bessel function of the second kind. It fulfils the differential equation:

$$z^{2}y''+zy'-\left( z^{2}+n^{2} \right)y=0$$

. (A16)

This function is implemented in many computer algebra systems (e.g. Mathematica: „BesselK(n,z)“ and Matlab: „besselk(nu,z)“).

Notice: A thin light line (small *w_P0_*) is practically accompanied with a high illumination divergence. In turn, the solution is only valid for a small field-of-view (consider *w_P0_* as a parameter).

# 4. Light sheet illumination

The illumination of a light sheet microscope is preferably shaped in a way, that only the in-focus plane of the sample is illuminated. The challenge is to form a thin and at the same time collimated illumination. Practically, elliptical Gaussian beams or lattice light-sheets [2] are employed. To exclude the compromise between sheet thickness and divergence from the discussion, we approximate the illumination by a collimated Gauss-sheet in xy‑plane with 2 · *w_P0z_* height. The intensity distribution of such a light sheet (propagating in x- or y-direction) can be described by:

$$I_{P}\left( x,y,z \right)=I_{P0}\cdot e^{-2\left( \frac{z}{w_{P0z}} \right)^{2}}$$

. (A17)

The signal factor (equation 7) calculates using the detection optics point spread function (equation 6) with optical axis in z-direction and the illumination distribution (equation A17):

$$J_{f}=\int e^{-2\left( \frac{z}{w_{P0z}} \right)^{2}}\iint{PSF}_{D}\left( x,y,z \right)dxdydz$$

. (A18)

The normalisation property of the detection optics point spread function (equation 5) results independently of *PSF_D_* in:

$$J_{f}=\int e^{-2\left( \frac{z}{w_{P0z}} \right)^{2}}dz$$

, (A19)

$$J_{f}=\sqrt{\frac{\pi}{2}}w_{P0z}$$

. (A20)

For light sheet microscopy *J_f_* can be understood as a measure of axial resolution.

Notice: A thin light sheet (small *w_P0z_*) is practically accompanied with a high illumination divergence. In turn, the solution is only valid for a small field-of-view (consider *w_P0z_* as a parameter).

# 5. Wide-field illumination

A wide-field microscope features a homogeneous illumination flux through the entire sample:

$$I_{P}\left( x,y,z \right)=I_{P0}$$

. (A21)

The signal factor (equation 7) using the detection optics point spread function (equation 6) with an optical axis in z-direction and the illumination distribution (equation A21) calculates to:

$$J_{f}=\iiint{PSF}_{D}\left( x,y,z \right)dxdydz$$

. (A22)

Since this expression is divergent, it leads to a finite result only for samples of a finite height ∆*z*. Using the normalisation property of the detection optics the point spread function (equation 5) results due to energy conservation independently of *PSF_D_* in:

$$J_{f}=\int_{0}^{\Delta z} \iint{PSF}_{D}\left( x,y,z \right)dxdydz=\Delta z$$

. (A23)

According to this the signal increases linearly with the sample height ∆*z* which was expected because a wide-field microscope does not feature optical sectioning. Thus, thick samples emit out of focus light which does not contribute to a sharp image, but to overall noise. To account for this, an additional in-focus signal factor is introduced. As a measure the depth of field of the wide-field microscope featuring the numerical aperture NA_D_ is used [3]:

$$J_{f}=2\frac{\lambda n}{{NA}_{D}^{2}}$$

. (A24)

# References

1. Mertz J. Introduction to Optical Microscopy. Cverton Y, editor. Greenwood Village, Colorado: Robert and Company Publishers; 2010.

2. Chen B-C, Legant WR, Wang K, Shao L, Milkie DE, Davidson MW, et al. Lattice light-sheet microscopy: Imaging molecules to embryos at high spatiotemporal resolution. Science (80- ). 2014;346: 12579981–125799812. doi:10.1126/science.1257998

3. Michael Bass, Decusatis C, Enoch J. Handbook of Optics, Volume I: Geometrical and Physical Optics, Polarized Light, Components and Instruments [Internet]. 3rd ed. Bass M, editor. Book. New York: Mcgraw Hill Book Co; 2009. doi:10.1036/007047740X
