# Supplementary material for: Thermal illumination limits in 3D Raman microscopy: A comparison of different sample illumination strategies to obtain maximum imaging speed
Source: PLoS One. 2019 Aug 13;14(8):e0220824. doi: 10.1371/journal.pone.0220824 (PMC6692011; doi:10.1371/journal.pone.0220824)
Supplement: S2 Appendix — (DOCX) [file pone.0220824.s002.docx]

Calculating *u_f_*(*T*) in case of no diffusion (S2)

In the absence of thermal diffusion (*k* = 0 e.g. for very short illumination or wide field illumination of large samples) we easily get the analytical solution of the heat equation with a volumetric heat source (equation 8 and 9):

$$u\left( \vec{r},T \right)=I_{P}\left( \vec{r} \right)\mu_{a}\frac{T}{\varrho c_{p}}+u_{0}\left( \vec{r} \right)$$

. (A1)

This solution is independent of the actual shape of the illumination. Without diffusion we solve equation A1 for *I_P_*(*r*) and get by identifying *u*(*r,T*) ≤ *u_crit_*:

$$I_{P0}\left( T \right)\leq\left( u_{crit}-u_{0} \right)\frac{\varrho c_{p}}{\mu_{a}T}$$

(A2)

Were *T* should be understood as a parameter. Due to linearity the solutions for all illumination geometries of *I_P0_*(*T*) can be normalised for the absorption coefficient *µ_a_* and the maximal allowed temperature rise (*u_crit_ - u_0_*). For this the comparable temperature factor *u_f_*(*T*) is introduced in the main text (equation 12). It does only depend on the illumination geometry, the illumination period *T* and the least possible number of material constants:

We get according to equation A2:

$$I_{P0}\left( T \right)\leq\frac{\left( u_{crit}-u_{0} \right)}{\mu_{a}}\cdot u_{f}\left( T \right)$$

(12)

were *u_f_*(*T*) is in case of no diffusion:

$$u_{f}\left( T \right)=\frac{\varrho c_{p}}{T}$$

. (13)

The actual absorption coefficient *µ_a_* and the actual temperature threshold *u_crit_* are not decisive for the comparison of different illumination geometries since they are independent of the utilised microscopy method.
